# A Comparative Evaluation of Point-of-Care and Laboratory HbA1c Testing in Diabetes Care: An Indian Perspective

**DOI:** 10.7759/cureus.69956

**Published:** 2024-09-22

**Authors:** Lokendra Rathod, Sameera Khan, Swasti Shubham, Niranjan Bisne, Sammradhi Singh, Manoj Kumar, Rajnarayan Tiwari, Devojit K Sarma

**Affiliations:** 1 Molecular Biology, Indian Council of Medical Research (ICMR) - National Institute for Research in Environmental Health, Bhopal, IND; 2 Biomolecular Engineering and Biotechnology, Rajiv Gandhi Proudyogiki Vishwavidyalaya, Bhopal, IND; 3 Pathology, People's College of Medical Sciences and Research Centre, Bhopal, IND; 4 Microbiology, Indian Council of Medical Research (ICMR) - National Institute for Research in Environmental Health, Bhopal, IND; 5 Epidemiology, Indian Council of Medical Research (ICMR) - National Institute for Research in Environmental Health, Bhopal, IND

**Keywords:** diabetes management, diagnostic accuracy, glycemic control and diabetes, hba1c, point-of-care test

## Abstract

Aim: This study aimed to assess the diagnostic performance of a point-of-care (POC) glycated hemoglobin (HbA1c) device in the Indian population against the standard laboratory (high-performance liquid chromatography {HPLC}) method for the effective management of diabetes in India.

Methods: This study on the diagnostic accuracy of a POC HbA1c device involved 121 participants. These participants were categorized into two groups according to their HbA1c levels - one group with HbA1c values below 6.5% and another group with HbA1c values equal to or greater than 6.5%. The HbA1c levels in enrolled participants were estimated using both the POC device (HemoCue HbA1c 501; Ängelholm, Sweden: HemoCue AB) and the standard HPLC-based method. The level of agreement and concordance between the two test results were assessed by the Bland-Altman plot and Lin’s concordance correlation coefficient. Sensitivity, specificity, diagnostic accuracy, positive likelihood, and negative likelihood ratios of POC-HbA1c device were assessed with a 6.5% HbA1c cut-off.

Results: The mean HbA1c values obtained by the two methods showed no statistically significant difference with a minimum effect size (Cohen's d = 0.035), indicating there was a negligible difference between these methods. The Bland-Altman plot revealed that most values were within acceptable limits (95% CI: -0.5 to 0.7) and Lin’s concordance correlation coefficient showed strong agreement (p < 0.0001). The POC-HbA1c device demonstrated an area under the curve (AUC) of 0.991 (95% CI: 0.953-1.000) with sensitivity, specificity, diagnostic accuracy, positive likelihood, and negative likelihood ratios of 93.62%, 97.30%, 95.87%, 34.64 and 0.07, respectively, compared to the standard diagnostic assay.

Conclusions: The diagnostic accuracy, sensitivity, and specificity demonstrated by the POC-HbA1c device to the standard HPLC method offers a viable and practical solution for diabetes management in India. Its ability to provide rapid and reliable results at the point of care can improve patient outcomes, reduce healthcare costs, and enhance access to diabetes care, especially in primary care, remote areas, and resource-limited settings of developing countries like India.

## Introduction

Type 2 diabetes mellitus (T2DM) is a multifactorial chronic metabolic disease that is rapidly achieving the status of a global epidemic. Globally, approximately 537 million people are estimated to have diabetes mellitus (DM), with 95% of these cases being T2DM. This accounts for a global prevalence of 10.5% as of 2021 [1]. India bears a substantial portion of this burden, with an estimated 101 million living with T2DM and an additional 136 million having pre-diabetes. The prevalence of T2DM and pre-diabetes in India was calculated to be 11.4% and 15.3%, respectively [1,2].

With such a huge burden, diabetic complications contribute to a considerable portion of morbidity and mortality globally. Undiagnosed or uncontrolled T2DM leads to several microvascular and macrovascular complications involving every major organ system, including stroke, coronary heart disease and heart failure, peripheral neuropathy, retinopathy, diabetic kidney disease, peripheral vascular disease, and increases susceptibility to infectious diseases and cancer [3]. Timely intervention and sustained glycemic control are essential for diabetes management to reduce medical complications and improve quality of life in the long term.

The diagnosis and prognosis of T2DM hinges on the status of blood glucose levels and their maintenance within the prescribed range, as assessed by frequent measurement of blood glucose and its surrogate, glycated hemoglobin (HbA1c) [4]. At present, HbA1c is used for both diagnosis of T2DM and monitoring of glycemic control, as it reflects the average plasma glucose levels over the previous eight to 12 weeks, thus obviating the need for repeated blood sampling. HbA1c measurement does not require any special preparation like fasting, nor has any diurnal variation. Given its reliability and predictive value, HbA1c is integral to the diagnosis and management of diabetes [5,6].

Several methods are available for HbA1c measurements, such as immunoturbidimetry, boronate affinity chromatography, ion-exchange high-performance liquid chromatography (HPLC), and enzymatic assays. These methods work on different principles based on either charge difference or structural difference. To maintain traceability and improve patient care, an HPLC-based method was developed as a reference measurement procedure and is considered the standard method for HbA1c measurement [7,8].

However, the limited availability and higher cost of HPLC-based HbA1c measurement are prohibitive, particularly in developing countries like India. These limitations also pose challenges in outreach programs and population-based research programs, where point-of-care (POC) would be more practical. Due to this, in 2021, the American Diabetes Association (ADA) advised clinicians to use POC for HbA1c measurement for timely medication and better glycemic control [9]. However, the reproducibility, precision, and accuracy of POC test procedures need to be rigorously evaluated to maintain clinical standards.

In the present study, we compare one such POC HbA1c measurement system (HemoCue HbA1c 501; Ängelholm, Sweden: HemoCue AB) with the standard HPLC measurement (HLC-723GX Automated Glycohemoglobin Analyzer; Tokyo, Japan: Tosoh) in enrolled participants in an urban setting of Central India. We aimed to assess the usefulness of this POC devise against the standard test in glucose monitoring and control, as this finding will pave the way for efficient screening and timely management of alarmingly increasing T2DM cases in India.

## Materials and methods

This population-based diagnostic accuracy study of a POC HbA1c device was conducted in the urban area of Bhopal, Madhya Pradesh, Central India, between November 2023 and April 2024. The sample size for diagnostic accuracy was estimated using the method described by Akoglu in 2022 [10]. This analysis used a power of 90% with a minimum expected sensitivity of the point-of-care (POC) device set at 90% and a significance level of 5%. The minimum sensitivity of the HPLC-based test was considered to be 75% at an HbA1c cut-off level of 6.5%, as reported by Chivese et al. [11]. The total sample size was found to be 123 at 95% confidence level. Health camps were organized for screening of T2DM in different institutional set-ups (universities, offices), and 121 eligible consented individuals within the age group 30-70 years were enrolled in the study. The diagnostic criteria (HbA1c level ≥6.5% for diabetes, ≥5.7% to <6.5% for pre-diabetes, and <5.7% for normal) defined by the American Diabetes Association were used to identify T2DM cases [5]. Demographic and anthropometric data were collected from all participants by using a standard questionnaire (appendix I). Informed written consent was obtained from all participants after briefing them on the study objectives and methodology.

Approximately 2 ml of venous blood was collected using K3 EDTA vacutainer by an experienced technician. The collected blood samples were immediately stored at 4°C and transported to standard laboratory for HbA1c measurement using the HPLC-based method. Fasting blood sugar levels were measured using the HemoCue Glucose 201 system. HbA1c was measured using both POC HbA1c measurement system (HemoCue HbA1c 501) and standard HPLC-based method (HLC-723GX Automated Glycohemoglobin Analyzer) on the same day. Both fasting blood glucose and POC-HbA1c measurements were carried out during blood collection. The HbA1c level was estimated using a standard HPLC-based method conducted in a laboratory accredited by the National Accreditation Board for Testing and Calibration Laboratories (NABL), which maintains international standards and stringent quality management. All tests were carried out strictly following the manufacturer’s protocol. Both tests were conducted and interpreted blinded to the participant’s HbA1c status, and participants were divided into two groups as HbA1c with ≥6.5% and HbA1c with <6.5%.

Statistical analyses for quantitative data, such as demographic and anthropometric data, were analyzed using mean and standard deviation (SD). An unpaired two-tailed t-test was used to test the significance in the mean differences of HbA1c between these two methods. To quantify the effect size of the mean differences between the two measurements, Cohen's d statistics was used [12]. The Bland-Altman plot and scatter plot were used to visualize the agreement in estimated HbA1c values by the two methods (mean of HbA1c estimates obtained from HemoCue HbA1c 501 system and HPLC vs. difference in HbA1c values between HemoCue HbA1c 501 system and HPLC test) [13]. To quantify the concordance between these two test methods, Lin’s concordance correlation coefficient (CCC) and Pearson’s correlation coefficient were estimated. The area under the receiver-operating characteristic (ROC) curve was used to estimate the accuracy of POC-HbA1c method against the standard HPLC method, using 6.5% as the cut-off level for HbA1c as determined by the HPLC-based method. The ROC curve was generated using MedCalc (Ostend, Belgium: MedCalc Software Ltd.) statistical software version 19.2.6. The POC method using the HemoCue HbA1c 501 system was compared to the standard laboratory method (HPLC) with a cut-off HbA1c level of 6.5% to determine its sensitivity, specificity, accuracy, and positive and negative likelihood ratios [5].

## Results

A total of 121 individuals participated in this study, consisting of 72 (59.5%) males and 49 (40.5%) females. The mean age of the study group was 46.51 ± 10.73 years (male: 44.15 ± 11.28; female: 48.02 ± 10.16), with a mean BMI of 25.97 ± 3.80 and mean fasting blood sugar (FBS) levels of 113 ± 41.92 mg/dL (Table 1).

**Table 1:** Demographic and anthropometric characteristics of the enrolled participants. The age, duration of diabetes, BMI, W/HtR, W/HR, and FBS values were represented in mean ± SD. BMI: body mass index; W/HtR: waist-to-height ratio; W/HR: waist-to-hip ratio; FBS: fasting blood sugar

Parameters	Cohort	Male	Female
Enrollment (N, %)	121 (100)	72 (59.5)	49 (40.5)
Diabetic (N, %)	61 (50.4)	44 (61.2)	17 (34.7)
Non-diabetic (N, %)	47 (38.9)	23 (31.9)	24 (48.9)
Pre-diabetic (N, %)	13 (10.7)	05 (6.9)	08 (16.4)
Age (years)	46.51 ± 10.73	44.15 ± 11.28	48.02 ± 10.16
Duration of diabetes (in months)	36.21 ± 53.68	71.61 ± 55.07	66 ± 62.13
BMI	25.97 ± 3.80	25.73 ± 3.47	26.35 ± 4.27
W/HtR	0.57 ± 0.06	0.55 ± 0.05	0.59 ± 0.08
W/HR	0.95 ± 0.08	0.96 ± 0.07	0.92 ± 0.08
FBS	113 ± 41.92	120 ± 48.08	100 ± 26.33

**Figure 1:**
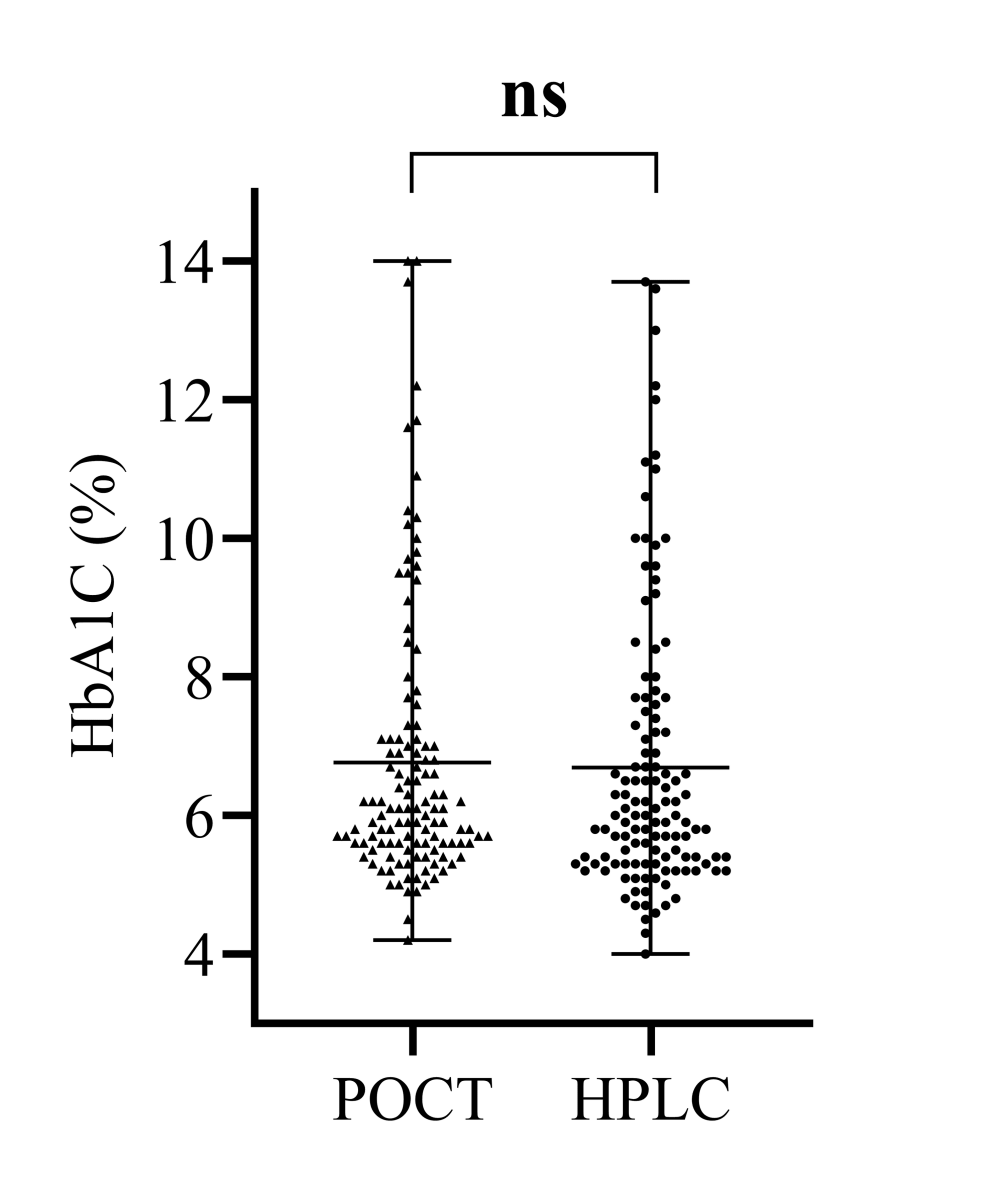
Mean and ranges of HbA1c values measured by the two methods. HbA1c: glycated hemoglobin; POCT: point-of-care testing; HPLC: high-performance liquid chromatography

HbA1c values were estimated by both the POC (HemoCue) and standard (HPLC)-based method for all the 121 participants. The mean of HbA1c values measured by HemoCue HbA1c 501 was 6.76% (SD ± 1.99; range = 4.20-14.00) and that for HPLC method was 6.69% (SD ± 2.04; range = 4.00-13.70). The differences in these two estimates were non-significant (t = -1.647, p = 0.10) with a minimum effect size (Cohen’s d = 0.035) suggesting a very small difference in the test results between the two methods. The details of HbA1c measurements by the two methods are presented in appendix II. Figure 1 shows the comparative distribution of HbA1c levels measured by both methods.

**Figure 2:**
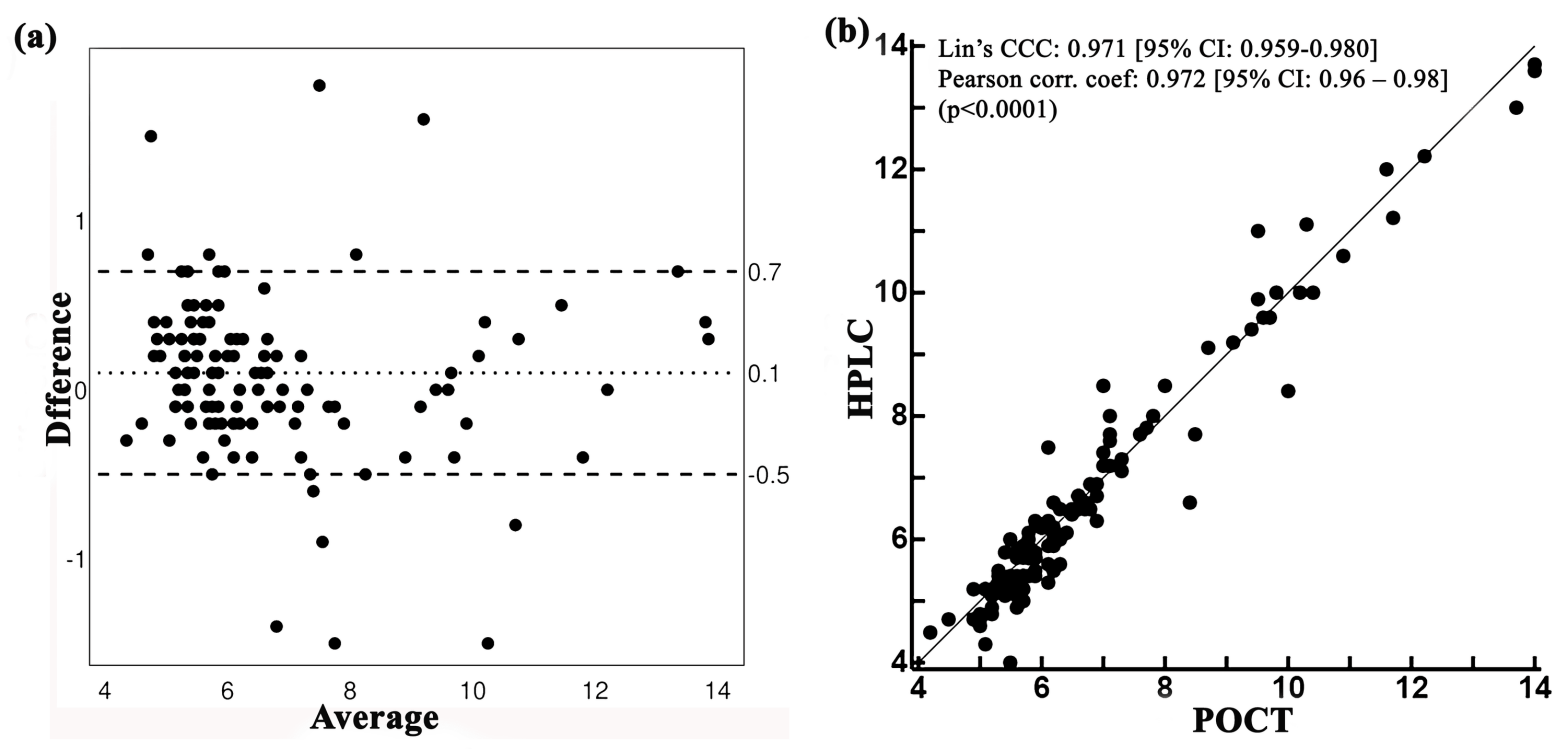
Agreement between the POCT-HbA1c using HemoCue HbA1c 501 system and standard diagnostic laboratory method. (a) Bland-Altman plot for visualizing the concordance between HbA1C levels estimated by two methods. (b) Scatter plot with Lin’s concordance correlation coefficient and Pearson’s correlation coefficient are also displayed along with their 95% confidence intervals. HemoCue HbA1c 501 system (Ängelholm, Sweden: HemoCue AB). HbA1c: glycated hemoglobin; POCT: point-of-care testing; HPLC: high-performance liquid chromatography

The Bland-Altman plot (difference between the HemoCue HbA1c 501 system and reference standard (HPLC) vs. the average HbA1c value by both methods) showed that a majority of the mean values were within the acceptable range of ±1 SD with a mean difference (bias) of 0.1 (Figure 2a). The HbA1c levels of 109 out of 121 participants analyzed with point-of-care test (HemoCue) and the standard diagnostic laboratory (HPLC) method were within the range of agreement limits (95% confidence intervals: -0.5 to 0.7), with only six upper outliers and six lower outliers outside the agreement limits range (Figure 2a). This indicates that 90.1% of the HbA1c measurements obtained from the HemoCue HbA1c 501 system are aligned with the standard laboratory method. Both Lin’s concordance correlation coefficient (CCC) (0.971; 95%CI: 0.959-0.980) and Pearson’s correlation coefficient (r = 0.972; 95% CI: 0.960-0.980) for HemoCue device indicated good concordance and substantial level of agreement with the standard laboratory method, with high significance (p < 0.0001) (Figure 2b).

**Figure 3:**
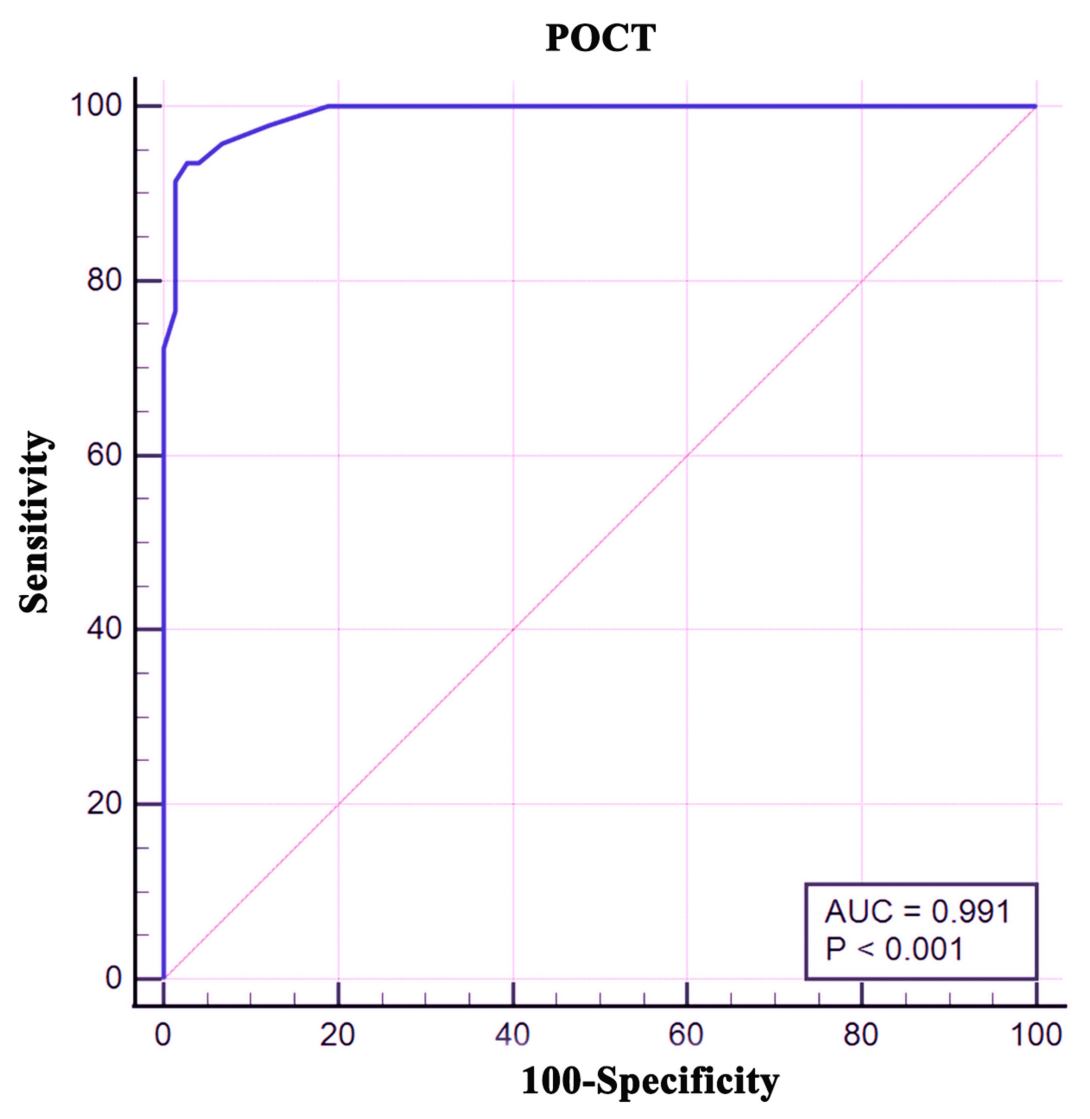
ROC curve derived AUC for POCT-HbA1c device (HemoCue HbA1c 501) against standard method with HbA1c cut off 6.5%. HemoCue HbA1c 501 (Ängelholm, Sweden: HemoCue AB) ROC: receiver operating characteristic; AUC: area under the curve; HbA1c: glycated hemoglobin; POCT: point-of-care testing

Receiver operating characteristic (ROC) curve-derived area under the curve (AUC) analysis for the point-of-care HemoCue HbA1c 501 device showed overall test performance with an AUC of 0.991 (95% CI: 0.953-1.000) (Figure 3).

The AUC indicates strong performance accuracy of the POC (HemoCue HbA1c 501 devise) in estimating HbA1c. Similarly, the high value (0.909) of Youden index J signifies an excellent performance of the POC test with a good trade-off between detecting positive cases of T2DM and avoiding false positives. To assess the clinical utility of POC-HbA1c, its HbA1c estimates were compared to standard laboratory results (HPLC method) using a cut-off point of 6.5%, as shown in Table 2.

**Table 2:** Sensitivity and specificity of the POCT-HbA1c system (HemoCue HbA1c 501) based on a 6.5% cut-off point. HemoCue HbA1c 501 (Ängelholm, Sweden: HemoCue AB) HbA1c: glycated hemoglobin; POCT: point-of-care testing; HPLC: high-performance liquid chromatography

HbA1c test by POCT	HbA1c test by standard HPLC-based method	
	HbA1c ≥ 6.5	HbA1c < 6.5
HbA1c ≥ 6.5	44	2
HbA1c < 6.5	3	72
Total	47	74

The POC-HbA1c test has a sensitivity of 93.62% and a specificity of 97.30%. POC-HbA1c showed a positive predictive value (PPV) of 95.65% for participants with HbA1c ≥ 6.5 and a negative predictive value (NPV) of 96.0% for those with HbA1c < 6.5. POC-HbA1c had a diagnostic accuracy (effectiveness) of 95.87% when expressed as a proportion of true positives and true negatives (participants correctly identified by POC-HbA1c). The positive likelihood ratio (LR+) and negative likelihood ratio (LR-) were found to be 34.64 and 0.07, respectively (Table 3).

**Table 3:** Diagnostic accuracy of point-of-care HbA1C (HemoCue HbA1c 501 device) measurement. HbA1c of 6.5% or greater is considered as a positive test indicating poor glycemic control. *Kappa statistics with a value of < 0.001 were considered significant. HemoCue HbA1c 501 (Ängelholm, Sweden: HemoCue AB) PPV: positive predictive value; NPV: negative predictive value; LR+: positive likelihood ratio; LR-: negative likelihood ratio; HPLC: high-performance liquid chromatography; POC: point-of-care

Comparison	POC vs. HPLC (n = 121)
Accuracy	95.87 (90.62-98.64)
Sensitivity	93.62 (82.46-98.66)
Specificity	97.30 (98.58-99.67)
Kappa	0.91* (0.84-0.99)
PPV	95.65 (84.84-98.86)
NPV	96.00 (88.92-98.63)
LR+	34.64 (8.81-136.19)
LR-	0.07 (0.02-0.20)

## Discussion

Glycated hemoglobin (HbA1c) remains the first choice of clinicians for the diagnosis and management of diabetes. Conversely, plasma glucose level, the most commonly used convenient indicator for patient’s glycemic control and compliance with treatment, is inconvenient due to the requirement of at least 8 hours of fasting and dietary intake dependency [14]. Since HbA1c reflects long-term blood sugar control and higher levels are linked to a greater risk of serious complications, such as heart disease, kidney problems, and vision issues, monitoring HbA1c is crucial for effective diabetes management [15]. However, the standard laboratory-based measurement methods (such as HPLC), although considered gold standards, have disadvantages, such as planned venipuncture and longer waiting times [16]. Validated point-of-care (POC) HbA1c testing devices, with their faster turnaround time and lack of need for specialized laboratory infrastructure, enable early diagnosis of pre-diabetes, enhance patient compliance, and allow for monitoring of patient prognosis and treatment adherence during a single visit. This is especially advantageous in remote rural and community settings [17].

The findings of this study demonstrate a substantial agreement between HbA1c measurement by POC-HbA1c measurement system (HemoCue HbA1c 501) and the standard HPLC measurement (HLC-723GX Automated Glycohemoglobin Analyzer). The mean difference between the POC-HbA1c measurements and the HPLC-based method was only 0.1, indicating that the POC-HbA1c measurements have a very small difference and align well with those measured by the standard diagnostic lab using the HPLC-based method. From a clinical point of view, this minute difference is not expected to affect the discrimination of a positive and negative sample. With only 12 outliers (9.9%), 109 out of 121 measurements (90.1%) by the POC-HbA1c method were within the 95% limit of agreement (Figures 2a, 2b, appendix I). Although the observed outlier is higher in the present study in comparison to that of Berbudi et al. (7.41%), this comes under the 95% confidence interval (4.6%-15.2%), indicating less role of the observed outliers in the limit of agreement between the two test results [18]. This level of agreement was also observed by Berbudi et al. in Indonesian populations [18]. A higher concordance (Lin’s CCC: 0.971, 95% CI: 0.959-0.980) was observed between the two test methods, with good diagnostic accuracy (95.9%), sensitivity (93.6%), and specificity (97.3%) of POC-HbA1c test method. Similar findings have been reported by other authors. Al Hayek et al. reported that the POC-HbA1c values were consistent with the standard lab HbA1c values (SD of bias = 0.55 and 95% CI = -0.78 to 1.4) and the agreement between the two methods was also higher (r = 0.798, 95% CI: 0.712-0861) [6]. The sensitivity (93.62%) and specificity (97.30%) of POC-HbA1c using the HemoCue HbA1c 501 system in this study were observed to be higher. Berbudi et al. when compared POC-HbA1c levels of HemoCue 501 system with standard lab test, reported a higher sensitivity (97.83%) but lower specificity (77.42%) [18]. The diagnostic accuracy, sensitivity, and specificity of the HemoCue device were reported to be 89.32, 90.63, and 87.18, respectively, by Khadanga et al. [19]. In populations with a low disease prevalence (<50%), a higher positive predictive value (PPV) and increased specificity enhance the likelihood of obtaining accurate positive results [20]. Consequently, the high specificity (97.30%) and positive predictive value (PPV) (95.65%) of the POC device observed in this study will aid clinicians in accurately diagnosing T2DM in countries like India, where the prevalence of T2DM is 11.4% [2]. Similarly, the observed higher positive likelihood ratio (34.64) and lower negative likelihood ratio (0.07) indicate the high reliability of the POC test for both diagnosing and excluding diabetes. In clinical settings, such a test would be highly beneficial for accurate and effective diabetes management as likelihood ratios are independent of population characteristics and can be used at the individual patient level [21]. This level of agreement indicates the utility of using POC-HbA1c (HemoCue HbA1c 501 system) for monitoring blood glucose levels, supporting the clinicians in managing the patients and improving diabetes therapy outcomes.

Estimation of HbA1c has become a standard of care test in case of diabetes management. This single test is useful in screening, diagnosis, and long-term management [5]. However, in the Indian context, limited availability and high cost make HbA1c testing prohibitive in peripheral and rural areas, compromising health care regarding T2DM. Point-of-care devices like HemoCue HbA1c 501 are portable, relatively low cost (both device cost and cost per test), easy to use with minimal training required, and have a very short turnaround time [18]. These devices have been shown to improve patient compliance and facilitate decision-making processes, thereby improving patient care [6,17]. Additionally, these advantages are valuable for population-based research. There is evidence that metabolic non-communicable diseases are increasing in India, especially in rural areas where health infrastructure for effective diagnosis and management of these conditions is scarce [2]. Thus, validation of POC devices like the HemoCue HbA1c 501 system in a broader range of socio-epidemiological settings (such as ethnic, age, and clinical groups) in India is essential to ensure its accuracy and reliability across diverse patient groups.

Our test subjects were middle-aged to elderly volunteers, and the tests were done for screening of T2DM; therefore, included normal healthy subjects, subjects with previously undiagnosed T2DM, and those with known T2DM who are on treatment. The sample used for HbA1c was venous blood for both test methods, while most previous studies used capillary blood for POC test. Good result concordance in venous blood may allow sample from a single venipuncture to be used for multiple tests including POC-HbA1c, making the procedure less invasive. Alternatively, capillary blood from a fingerpick may be used where venipuncture is not required. The diagnostic accuracy of POC HbA1c device study demonstrated promising results with adequate sample size, ensuring reliable data. The device’s ability to detect even minimal differences in HbA1c levels underscores its sensitivity, high predictive capability, and significant likelihood ratios further solidifying its potential as a valuable diagnostic tool. These findings suggest that the POC HbA1c device can accurately measure HbA1c levels, offering a convenient and efficient option for clinical practice. However, to fully establish its reliability and applicability, further validation in various settings such as different ethnic backgrounds, age groups, and geographic regions with varying T2DM prevalence rates is essential.

## Conclusions

To conclude, we find that the POC-HbA1c measurement system (HemoCue HbA1c 501) has significant potential for effective diabetes management, especially in areas with limited resources. The results show a high degree of concordance between POC-HbA1c measurements and standard HPLC method with minimal mean difference and substantial agreement within the 95% limit. There was good diagnostic accuracy, sensitivity, and specificity for the POC (HemoCue HbA1c 501) system as compared to a conventional laboratory test method. This indicates that POC-HbA1c has advantages in terms of portability, cost-effectiveness, user-friendliness, and quick turnaround time that make them extremely useful in rural or community setups where accessibility to laboratories is not available. Therefore, in this respect, this study confirms the validity of the HemoCue HbA1c 501 system in India by indicating its capacity to enhance screening for diabetes, diagnosis, and long-term management of diabetes.

Overall, using POC-HbA1c devices in diabetes care can lead to increased patient adherence rates, and enable timely clinical decision-making thus improving health outcomes among diabetics, especially in those from poor backgrounds. More studies on wider implementation trials as well as continuous validation are necessary to strengthen robustness.

## Figures and Tables

**Figure 4:**
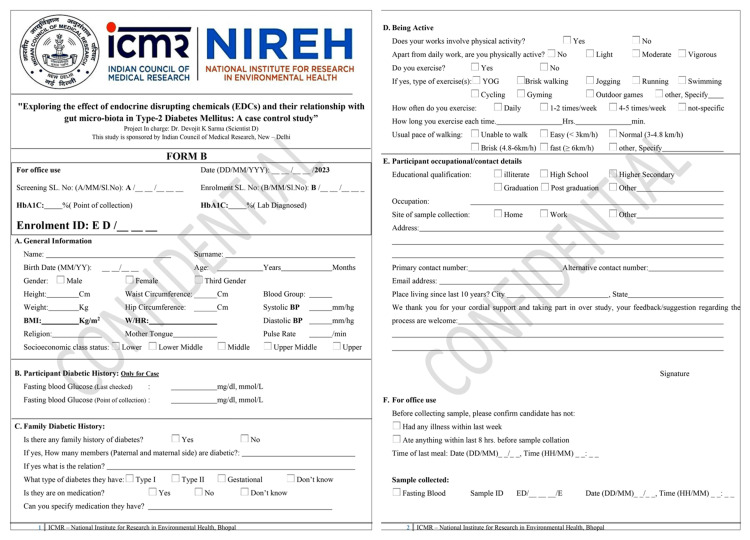
Questionnaire used for the collection of demographic, anthropometric, and health data from the enrolled individuals.

## Appendices

Appendix I

Appendix II

**Table 4:** Comparison of HbA1c measured by POCT-HbA1c (HemoCue HbA1c 501 system) and standard laboratory method (HPLC). HbA1c: glycated hemoglobin; POCT: point-of-care testing; HPLC: high-performance liquid chromatography

S. no.	ID	HemoQue	HPLC	Difference	Mean
1	E001	6.1	5.9	-0.2	6
2	E002	6.2	5.9	-0.3	6.05
3	E003	5.7	5.4	-0.3	5.55
4	E004	5.4	5.2	-0.2	5.3
5	E005	5.9	5.5	-0.4	5.7
6	E006	7.3	7.3	0	7.3
7	E007	8.5	7.7	-0.8	8.1
8	E008	6.2	6	-0.2	6.1
9	E009	5.7	5	-0.7	5.35
10	E010	5.2	5.2	0	5.2
11	E011	5.6	5.3	-0.3	5.45
12	E012	5.8	5.4	-0.4	5.6
13	E013	5.6	5.8	0.2	5.7
14	E014	5.6	5.8	0.2	5.7
15	E015	5.8	5.9	0.1	5.85
16	E016	6.1	6.2	0.1	6.15
17	E017	4.9	5.2	0.3	5.05
18	E018	5.3	5.3	0	5.3
19	E019	5.4	5.2	-0.2	5.3
20	E020	5.4	5.8	0.4	5.6
21	E021	5.6	4.9	-0.7	5.25
22	E022	5.7	5.8	0.1	5.75
23	E023	5.3	5.4	0.1	5.35
24	E024	5.5	6	0.5	5.75
25	E025	6.5	6.5	0	6.5
26	E026	5.6	5.4	-0.2	5.5
27	E027	5.9	6.3	0.4	6.1
28	E028	6.2	6.6	0.4	6.4
29	E029	5.3	5.5	0.2	5.4
30	E030	12.2	12.2	0	12.2
31	E031	6.3	5.6	-0.7	5.95
32	E032	5.5	5.4	-0.1	5.45
33	E033	5.4	5.1	-0.3	5.25
34	E034	5.1	5.2	0.1	5.15
35	E035	5.9	5.4	-0.5	5.65
36	E036	5	4.7	-0.3	4.85
37	E037	5.6	5.7	0.1	5.65
38	E038	5.6	5.2	-0.4	5.4
39	E039	5.9	5.8	-0.1	5.85
40	E040	6.4	6.1	-0.3	6.25
41	E041	6.9	6.3	-0.6	6.6
42	E042	5.7	5.4	-0.3	5.55
43	E043	5.6	5.1	-0.5	5.35
44	E044	6.7	6.6	-0.1	6.65
45	E045	5.4	5.3	-0.1	5.35
46	E046	4.5	4.7	0.2	4.6
47	E047	9.6	9.6	0	9.6
48	E048	7	7.4	0.4	7.2
49	E049	5.9	5.7	-0.2	5.8
50	E050	5.1	5.2	0.1	5.15
51	E051	5.7	5.9	0.2	5.8
52	E052	5.8	6.1	0.3	5.95
53	E053	5.2	5.1	-0.1	5.15
54	E054	8.7	9.1	0.4	8.9
55	E055	10.3	11.1	0.8	10.7
56	E056	6.1	5.6	-0.5	5.85
57	E057	4.9	4.7	-0.2	4.8
58	E058	7.1	7.6	0.5	7.35
59	E059	7.1	7.7	0.6	7.4
60	E060	5.1	4.3	-0.8	4.7
61	E061	5.6	5.3	-0.3	5.45
62	E062	5	4.8	-0.2	4.9
63	E063	9.5	9.9	0.4	9.7
64	E064	5	4.6	-0.4	4.8
65	E065	5.2	4.8	-0.4	5
66	E066	6.2	5.5	-0.7	5.85
67	E067	6.3	6	-0.3	6.15
68	E068	4.2	4.5	0.3	4.35
69	E069	5.2	4.9	-0.3	5.05
70	E070	6.1	5.3	-0.8	5.7
71	E071	6.8	6.5	-0.3	6.65
72	E072	9.7	9.6	-0.1	9.65
73	E073	11.6	12	0.4	11.8
74	E074	7	7.2	0.2	7.1
75	E075	11.7	11.2	-0.5	11.45
76	E076	6.6	6.7	0.1	6.65
77	E077	6.5	6.4	-0.1	6.45
78	E078	7.1	7.2	0.1	7.15
79	E079	10.2	10	-0.2	10.1
80	E080	9.1	9.2	0.1	9.15
81	E081	6.9	6.9	0	6.9
82	E082	8.4	6.6	-1.8	7.5
83	E083	6.3	6.5	0.2	6.4
84	E084	7.1	8	0.9	7.55
85	E085	5.5	4	-1.5	4.75
86	E086	5.3	5.3	0	5.3
87	E087	10.9	10.6	-0.3	10.75
88	E088	9.5	11	1.5	10.25
89	E089	5.4	5.3	-0.1	5.35
90	E090	9.4	9.4	0	9.4
91	E091	7	8.5	1.5	7.75
92	E092	7.3	7.1	-0.2	7.2
93	E093	7.6	7.7	0.1	7.65
94	E094	6.8	6.9	0.1	6.85
95	E095	6.9	6.7	-0.2	6.8
96	E096	10	8.4	-1.6	9.2
97	E097	13.7	13	-0.7	13.35
98	E098	14	13.6	-0.4	13.8
99	E099	14	13.7	-0.3	13.85
100	E100	5.7	5.7	0	5.7
101	E101	5.8	5.7	-0.1	5.75
102	E102	7.7	7.8	0.1	7.75
103	E103	6	6.2	0.2	6.1
104	E104	9.8	10	0.2	9.9
105	E105	5.9	5.7	-0.2	5.8
106	E106	5.8	5.7	-0.1	5.75
107	E107	5.6	5.8	0.2	5.7
108	E108	6.1	7.5	1.4	6.8
109	E109	5.8	6	0.2	5.9
110	E110	6	6.2	0.2	6.1
111	E111	6.1	6.3	0.2	6.2
112	E112	6.6	6.5	-0.1	6.55
113	E113	7.8	8	0.2	7.9
114	E114	8	8.5	0.5	8.25
115	E115	10.4	10	-0.4	10.2
116	E116	5.6	5.1	-0.5	5.35
117	E117	6.7	6.5	-0.2	6.6
118	E118	6.2	6.2	0	6.2
119	E119	5.7	5.2	-0.5	5.45
120	E120	5.3	5.3	0	5.3
121	E121	6.6	6.7	0.1	6.65
